# Label-Free Biosensor Based on Particle Plasmon Resonance Coupled with Diffraction Grating Waveguide

**DOI:** 10.3390/s24175536

**Published:** 2024-08-27

**Authors:** Wei-Ting Hsu, Yu-Cheng Lin, Huang-Chin Yang, Devesh Barshilia, Po-Liang Chen, Fu-Chun Huang, Lai-Kwan Chau, Wen-Hsin Hsieh, Guo-En Chang

**Affiliations:** 1 Department of Chemistry and Biochemistry and Center for Nano Bio-Detection, National Chung Cheng University, Chiayi 62102, Taiwan; walden966@yahoo.com.tw (W.-T.H.); ponskimo@gmail.com (H.-C.Y.); 2 Department of Mechanical Engineering and Advanced Institute of Manufacturing with High-Tech Innovations, National Chung Cheng University, Chiayi 62102, Taiwan; s60105s@hotmail.com (Y.-C.L.); barshiliadevesh@gmail.com (D.B.); p259872@gmail.com (P.-L.C.); fuchunghuang@hotmail.com (F.-C.H.)

**Keywords:** label-free biosensing platform, particle plasmon resonance, UV-assisted embossing, diffraction grating, gold nanoparticle surface

## Abstract

Particle plasmon resonance (PPR), or localized surface plasmon resonance (LSPR), utilizes intrinsic resonance in metal nanoparticles for sensor fabrication. While diffraction grating waveguides monitor bioaffinity adsorption with out-of-plane illumination, integrating them with PPR for biomolecular detection schemes remains underexplored. This study introduces a label-free biosensing platform integrating PPR with a diffraction grating waveguide. Gold nanoparticles are immobilized on a glass slide in contact with a sample, while a UV-assisted embossed diffraction grating is positioned opposite. The setup utilizes diffraction in reflection to detect changes in the environment’s refractive index, indicating biomolecular binding at the gold nanoparticle surface. The positional shift of the diffracted beam, measured with varying refractive indices of sucrose solutions, shows a sensitivity of 0.97 mm/RIU at 8 cm from a position-sensitive detector, highlighting enhanced sensitivity due to PPR–diffraction coupling near the gold nanoparticle surface. Furthermore, the sensor achieved a resolution of 3.1 × 10^−4^ refractive index unit and a detection limit of 4.4 pM for detection of anti-DNP. The sensitivity of the diffracted spot was confirmed using finite element method (FEM) simulations in COMSOL Multiphysics. This study presents a significant advancement in biosensing technology, offering practical solutions for sensitive, rapid, and label-free biomolecule detection.

## 1. Introduction

In recent years, interest in developing new methods and devices for offering simple, label-free, real-time, and highly sensitive sensing schemes for the analysis of biomolecular interactions has burgeoned. Surface plasmon resonance (SPR) spectroscopy is extensively utilized for studying interfacial phenomena by measuring minute changes in the charge density waves of free electrons that propagate along the boundary between a dielectric and a thin metal film [1]. SPR is not intrinsically resonant and can only be excited using an attenuated total reflection (ATR) optical setup. Standard SPR biosensors are based on the Kretschmann configuration [1], which is expensive, bulky, and difficult to miniaturize.

Particle plasmon resonance (PPR), also known as localized surface plasmon resonance (LSPR), is an intrinsic resonance of electron waves in metal nanoparticles that can be excited without the need for an ATR optical setup [2]. This resonance condition allows for the detection of rapid changes in the refractive index of the surrounding medium [2,3], as well as biomolecular interactions at the interface between nanoparticles and the solution [3,4]. Therefore, it is feasible to fabricate small sensors based on the PPR technique using a straightforward optical setup. PPR has been utilized previously for real-time monitoring of biomolecular interactions using array-based platforms [3,5] and optical fiber-based configurations [6,7,8,9,10,11].

Diffraction grating waveguides have been employed in various methods to monitor bioaffinity adsorption by coupling the transducer with out-of-plane illumination without additional coupling structures [12,13,14]. These diffraction grating waveguides are known by various names, including guided-mode resonance gratings, resonant waveguide gratings, and resonant grating waveguides. Recently, gratings have also been applied to bimodal waveguide interferometric sensors [15], common-path interferometric sensors [16], and side-wall grating sensors with box-like resonance shape [17]. A common biosensing approach is to couple a diffraction grating waveguide with an SPR sensor [18,19,20,21,22,23]. Gold nanoparticles (AuNPs) have also been employed in an SPR sensor to enhance sensitivity [24]. However, the feasibility of coupling a diffraction grating waveguide with PPR to develop biomolecular detection schemes has not been reported.

In this study, we present a highly sensitive label-free optical biosensor employing a subwavelength diffraction grating waveguide that simultaneously couples light into a plasmonic grating resonant mode and disperses the diffracted light for readout. Hence, this method is based on PPR without bulky optics. Due to the coupling of PPR with diffraction, the angle of the diffracted beam becomes extremely responsive to changes in the refractive index (RI) of the surrounding environment near the AuNP surface, as well as to biomolecular interactions occurring on the surface of the AuNP. To the best of our knowledge, this represents the inaugural effort in developing a label-free, real-time biosensor based on coupling a diffraction grating waveguide with PPR optical properties.

## 2. Materials and Methods

### 2.1. Reagents and Materials

The following chemicals were used as received: hydrogen tetracholoaurate(III) tetrahydrate (Showa), sodium borohydride (Showa), *n*-hexadecyltrimethylammonium bromide (CTAB, Acros, Antwerpen Belgium), 3-(mercaptopropyl)-trimethoxysilane (MPTMS, Acros), titanium(IV) n-butoxide [Ti(OBu)_4_, Acros], methacryloxypropyl-trimethoxysilane (MAPTMS, Gelest, Taipei, Taiwan), methacrylic acid (MAA, Lancaster, Taipei, Taiwan), butanol (RDH), sucrose (Hayashi), biotin (Sigma-Aldrich, Taipei, Taiwan), cystamine (Acros), dinitrophenyl-ε-aminocaproic acid (DNP, ICN), anti-dinitrophenyl antibody (anti-DNP, Sigma-Alrich), streptavidin (Sigma-Aldrich), anti-human serum albumin antibody (anti-HSA, Sigma-Aldrich), 1-ethyl-3-(3-dimethylaminopropyl)-carbodiimine hydrochloride (EDC, Fluka, Taipei, Taiwan), N-hydroxy-succinimide (NHS, Fluka), 2-(4-(2-hydroxyethyl)-1-piperazinyl)-ethanesulfonic acid (HEPES, Fluka). Surface functionalization solutions were prepared by dissolving 0.0122 g of biotin or 0.001 g of DNP, 0.192 g of EDC, and 0.02877 g of NHS in 10 mL of 0.01 M HEPES buffer (pH 7.4) and shaken for 30 min. Phosphate-buffered saline (PBS, pH = 7.4) was prepared by dissolving 2.1915 g of NaCl, 0.07455 g of KCl, 0.725 g of Na_2_HPO_4_·12H_2_O, 0.05 g of KH_2_PO_4_ in 250 mL of water. Poly(methyl methacrylate) (PMMA) was purchased from Sheng-Yang Business (Changhua, Taiwan) to manufacture the microfluidic chips. Preparation of all aqueous solutions was carried out using ultrapure water obtained from a Millipore Milli-Q water purification system (Millipore, Taipei, Taiwan), ensuring a specific resistance of 18.2 MΩ-cm. To achieve solutions with varying refractive indices (ranging from 1.342 to 1.403), sucrose solutions were dissolved in purified water at concentrations ranging from 6.8% to 41.7% [10]. 

### 2.2. Fabrication of Sensor Chips

**Figure 1:**
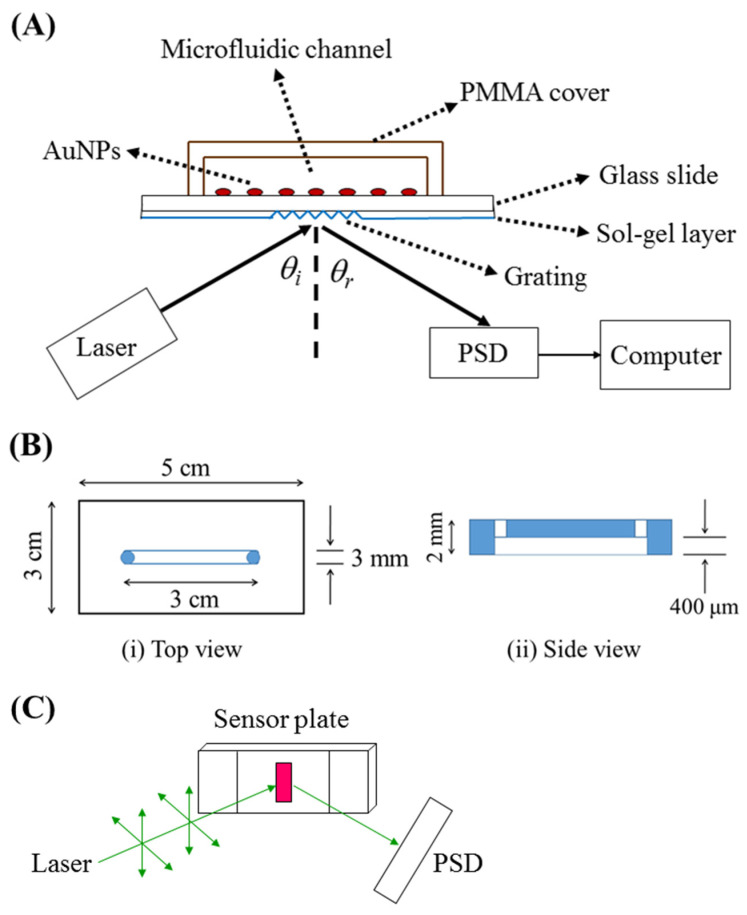
(**A**) Experimental setup for the grating-coupled PPR sensing system and schematic representation of the grating-coupled PPR sensor chip structure: (**B**) Design of the PMMA cover plate from (**i**) the top view and (**ii**) the side view; (**C**) Schematic of the optical train in the sensing system.

The sensor chip, as shown in Figure 1A, is composed of two plates: a PMMA cover plate (5 cm × 3 cm) with a microfluidic channel (~36 μL, length = 30 mm, width = 3 mm, depth = 0.4 mm) and a sensor plate with AuNPs immobilized on one side of a glass slide and a grating coupler on the opposite side of the glass slide (50 μm or 1 mm) which was sol–gel coated. The sample inlet and outlet for introduction were connected to two small access holes on the PMMA cover plate, which were mechanically drilled into the microfluidic channel. The design of the PMMA cover plate is shown in Figure 1B. The cover plate and sensor plate were bonded by coating UV glue (No. 9046, Everwide Chemical, Yunlin, Taiwan) on the cover plate, pressing against the sensor plate. Subsequently, the whole chip was exposed to UV light (VL-230BLB, λ = 365 nm, Vilber Lourmat, Taichung, Taiwan) for 10 min. A photograph of the microfluidic sensor chip is shown in Figure S1.

### 2.3. Sensing System and Measurements

The sensing system consisted of a green laser (532 nm, GCL-050-L-0.2%, CrystaLaser, Taoyuan, Taiwan), a microfluidic sensor chip on a rotation stage (06DTS-3M, Unice E-O Series, Taoyuan, Taiwan), a position-sensitive detector (PSD, 1L10, ON-TARK Photonics, Taoyuan, Taiwan) or a charge-coupled device (D80 camera, Nikon, Tokyo, Japan), as shown in Figure 1A. The optical train in the sensing system is shown in Figure 1C. The sensor’s performance was assessed by passing samples through the microfluidic channel on the sensor chip. During testing, the sample flow ceased once the channel was fully filled, and real-time sensor responses were monitored. Typically, several hundred seconds of sensor responses were used to establish a calibration curve.

### 2.4. Preparation of AuNPs

AuNPs were prepared as previously described [25] with minor modifications as per the following procedures. An aqueous solution containing hydrogen tetrachloroaurate (1.78 mL, 2.43 mM), chloroform (8.22 mL), and 0.0728 g of CTAB was mixed and stirred for 10 min to prepare the hydrogen tetrachloroaurate solution. Subsequently, a freshly prepared NaBH_4_ ethanol solution (0.8 mL, 0.15 M) was added to the hydrogen tetrachloroaurate solution under vigorous stirring. After stirring the solution for an additional 30 min, a ruby-colored organic phase containing AuNPs formed at the bottom. Further separation of the organic phase was then performed by placing the solution in a separation funnel for 2 h. The AuNP solution absorption spectra were measured using a Jasco V-570 spectrophotometer (Jasco, Easton, MD, USA). A characteristic absorbance peak was detected at 524 nm, depicted in Figure S2. Transmission electron microscopy (TEM) images of the AuNPs were acquired using a JEOL 1200 EX microscope (JEOL, Tokyo, Japan) operating at 120 kV. TEM samples were prepared by placing the sample on a copper grid and air-dried. Analysis of TEM images yielded histograms indicating that the average diameter of AuNPs was 10.1 ± 1.7 nm (*n* = 100), as shown in Figure S3.

### 2.5. Immobilization of AuNPs on Glass Slides

One side of each slide was exposed to 1% MPTMS in toluene for 12 h. After thorough rinsing, the slide was immersed in an AuNP solution for 12 h to form a self-assembled sub-monolayer of AuNPs on the surface. Subsequently, the AuNP-modified glass slide was sequentially rinsed with methanol and water and dried using a nitrogen purge.

### 2.6. Functionalization of Gold Nanoparticle Surface

To modify the surface of AuNPs with either DNP or biotin, we first formed a self-assembled monolayer (SAM) of cystamine (0.02 M). This process included introducing an aqueous solution of cystamine dihydrochloride into the sensor chip and allowing it to undergo reaction for 2 h at room temperature. Subsequently, the cystamine-modified AuNP surface was further functionalized with DNP or biotin using a surface functionalization solution containing the respective recognition molecule. The reaction proceeded for 2 h at room temperature, followed by rinsing with ultrapure water and storage in PBS until use.

### 2.7. Preparation and Characterization of Photopolymerizable Sol–Gel Films

The coating material was synthesized using Ti(OBu)_4_, MAPMTS, and MAA. Solution I was prepared by mixing 375 μL of MAPMTS with 57.6 μL of 0.02 M HCl and stirring for 30 min. Solution II was prepared by mixing 271 μL of Ti(Obu)_4_, 67 μL of MAA, and 72 μL of butanol and stirring for 30 min. Subsequently, solutions I and II were mixed and stirred for 30 min, and 56.8 μL of water was added and stirred for another 30 min to form solution III. Subsequently, 0.04 g of Irgacure 1800 (Ciba) was added to solution III and stirred for 2 h. The mixture was spin-coated onto a clean glass slide at 3000 rpm for 1 min to create film I. Subsequently, the sol–gel material underwent curing at 70 °C for 30 min and exposure to UV light (Mineralight UVG-54, UVP LLC, East Lyme, CT, USA) for 10 min. Film II was obtained through a hard bake at 110 °C for 12 h. The sol–gel films underwent analysis using several techniques: Infrared spectra were acquired using a Fourier transform infrared spectrometer (FT-IR 460 Plus, Jasco, Tokyo, Japan). Film thicknesses were determined via field-emission scanning electron microscopy (Hitachi 4800I, Hitachi, Tokyo, Japan). The RI of the sol–gel films was measured using a spectroscopic ellipsometer (SpecEI 2000, J.A. Wollam, Lincoln, NE, USA). Waveguide-propagating loss in the films was assessed using digital photography with the charge-coupled device (CCD) array of a Nikon D80 camera.

In this study, the planar waveguides were photopolymerizable sol–gel films on glass slides. These waveguides were mounted on a rotary stage, and light from a green laser (532 nm, GCL-050-L-0.2%, CrystaLaser) was coupled to the planar waveguide using a prism. The intensity decay of the guided mode in the direction of propagation was digitized and plotted in decibels (dB) concerning the propagation distance, *x*, of the steak line in cm unit [26,27]. The attenuation curves were analyzed using least squares regression 10 × log[(I(*x*)/I_0_] = −α × *x* + C, where I(*x*) denotes the average pixel intensity as a function of *x*, I_0_ denotes the average pixel intensity at *x* = 0, α denotes the loss coefficient in dB per cm, and C denotes a constant. The slope represents the propagation loss of the waveguide (dB/cm).

### 2.8. Fabrication of Grating

To fabricate the grating coupler on the sensor plate by UV-assisted embossing, a polydimethylsiloxane (PDMS) stamp was first produced by curing the liquid PDMS precursors (10 parts of Sylgard 184 prepolymer to one part of the curing agent) on a holographic glass grating (Edmund NT43-215, 1200 groves/mm, i.e., 833 nm) at 110 °C for 2 h. The depth of the master grating was measured to be approximately 130–150 nm by atomic force microscopy (AFM, SEIKO SPA-400, Tokyo, Japan). After detaching the PDMS stamp from the holographic glass grating, the PDMS stamp as shown in Figure S4 contained a grating structure with a period of 800 nm and a depth of 130.9 nm, as determined by AFM (shown in Figure S5). The PDMS stamp was cut to a size of 5 mm × 5 mm for subsequent application. The grating coupler was then fabricated by pressing the PDMS stamp onto sol–gel film I on the sensor plate, and the entire set was cured at 70 °C for 30 min, and then it was exposed to UV light (Mineralight UVG-54, UVP LLC) for 10 min. Subsequently, the PDMS stamp was removed, and the sensor plate was rinsed with butanol and subjected to a hard baking process at 110 °C for 12 h. As determined by AFM (shown in Figure S6), the final PDMS grating structure exhibited a period of 823 nm and a depth of 121.6 nm. The period of the sol–gel grating was further confirmed by the FE-SEM image (Hitachi 4800I) as shown in Figure S7. The sol–gel layer integrated with the grating acts as a grating waveguide.

## 3. Results and Discussion

### 3.1. Characterization of Photopolymerizable Sol–Gel Films

**Figure 2:**
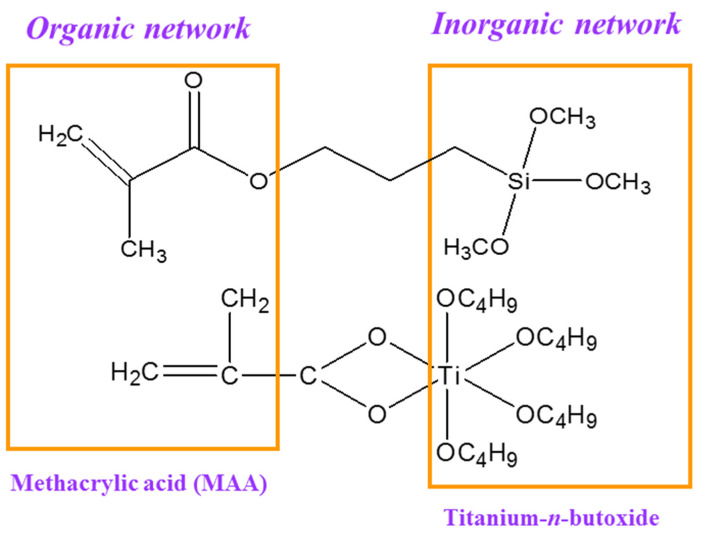
Schematic showing the formation of a hybrid organic/inorganic network via sol–gel reactions and photopolymerization.

As shown in Figure 2, the sol–gel films used to create gratings were produced by the hydrolytic condensation reaction of two precursors: titanium(IV) n-butoxide and methacryloxypropyltrimethoxysilane (MAPTMS). This process also involved forming an organic network through photopolymerization [28,29]. Methacrylic acid (MAA) was added to form a stable complex with the titanium precursor under hydrolytic conditions, thus enabling the incorporation of the TiO_2_ clusters into the matrix of silane polycondensates [30]. Irgacure 1800 (Ciba, Switzerland) was used as the photoinitiator for radical polymerization [31].

Figure S8 displays the FTIR spectra of the sol–gel film before and after UV light exposure. The characteristic absorption band at 1635 cm^−1^ corresponds to the stretching vibration of C=C in both MAPTMS and MAA, while the band at 1716 cm^−1^ is attributed to the stretching vibration of C=O in MAPTMS. Following UV exposure, the percent transmittance (%T) at 1635 cm^−1^ decreased notably, and the band at 1716 cm^−1^ shifted to 1724 cm^−1^, indicating photopolymerization within the film. The refractive index (RI) of the sol–gel films, determined using spectroscopic ellipsometry, was found to be 1.547 at 532 nm. Additionally, the film thickness, as observed from the cross-sectional SEM micrographs (Figure S9), was approximately 2.7 µm.

To examine the suitability of the photopolymerizable sol–gel films as a planar waveguide material, the waveguide propagation loss is a quantitative index of light energy loss in the waveguide [26,27]. From the plots of intensity decay versus propagation distance, as shown in Figure S10, the average waveguide propagation loss is estimated to be 0.041 ± 0.004 dB/cm (*n* = 2), which is comparable to or lower than that of many planar waveguide materials [26,27,32,33].

### 3.2. Optimization of Incident Angle and PSD Position

Figure 1A shows the optical setup of the grating-coupled PPR sensing system and the structure of the grating-coupled PPR sensor chip. A laser beam of 532 nm was incident on the grating of the glass slide (thickness, t = 1 mm or 50 μm) to excite the localized surface plasmon modes at an incident angle *θ_i_*. When *θ_i_* is within the coupling range, the incident light couples into and propagates in the waveguide and then exits the waveguide at the end face. A blank sample was used to test the optimum coupling angle at which the highest coupling intensity exiting the end face of the waveguide was measured. In this study, the laser beam was coupled into the glass slide at the optimum *θ_i_* = ~20°.

The position of the diffracted beam with varying angle *θ_r_* was measured using a PSD at a distance from the glass slide. One possible sensing mechanism is attributed to the Goos–Hanchen shift (GHS), which refers to the lateral shift between the actual observed and geometrical optics predicted reflected beams under total internal reflection. The principle of GHS has been proposed for SPR sensors [34,35]. The PSD distance was optimized by evaluating the refractive index sensitivity (RIS) and sensor resolution (SR) of the grating-coupled PPR sensor chip (glass slide thickness = 50 μm) at several distances. The RIS is defined as the slope (m) of the plot of signal versus refractive index unit (RIU) of samples. The SR refers to the smallest detectable change in RIU by the sensor and is calculated as σ/m, where σ represents the standard deviation of the system noise. To determine the RIS of our sensor, we measured the position of the diffracted beam in the PSD by exposing the bare AuNP surface to sucrose solutions with various refractive indices. When the concentration, and therefore the RI of a sucrose solution, increased within the range of 1.34–1.41, the position of the diffracted beam at PSD showed a linear shift with RISs of 0.77 mm/RIU, 0.97 mm/RIU, and 4.96 mm/RIU for PSD distances of 1 cm, 8 cm, and 15 cm, respectively. Although a longer PSD distance appears to yield a higher RIS, the noise also increases with increasing PSD distance. Therefore, the SRs at the three PSD distances were calculated. The results show that the SRs are 6.3 × 10^−4^ RIU, 2.1 × 10^−4^ RIU, and 5.7 × 10^−4^ RIU at PSD distances of 1 cm, 8 cm, and 15 cm, respectively. Although the SR did not vary significantly, the optimal SR was achieved at a PSD distance of 8 cm. Hence, the PSD was set at a distance of 8 cm from the glass slide.

### 3.3. Effect of Glass Slide Thickness on Sensitivity

To evaluate the sensitivity of our sensor concerning the thickness of the glass substrate, we compared the SRs of the grating-coupled PPR sensor chip using two glass slide substrates of different thicknesses. Results show that the SRs are 7.8 × 10^−4^ RIU and 3.1 × 10^−4^ RIU for the sensor chip using 1 mm and 50 μm substrates, respectively. This suggests that the sensor chip with a thicker substrate is inferior to that with a thinner substrate. Unfortunately, thin glass slide substrates are fragile, making chip production more difficult. In a control experiment with the same sensor chip but without AuNPs on the glass slide, the RIS in mm/RIU of the sensor decreased by approximately 6-fold.

We also tested the feasibility of the sensing platform detecting the intensity change instead of the position shift using 50 μm glass slides. The results show that the SR obtained by measuring the intensity change is 6.0 × 10^−4^ RIU, which is slightly lower than that obtained by measuring the position shift.

### 3.4. Theoretical Simulations

**Figure 3:**
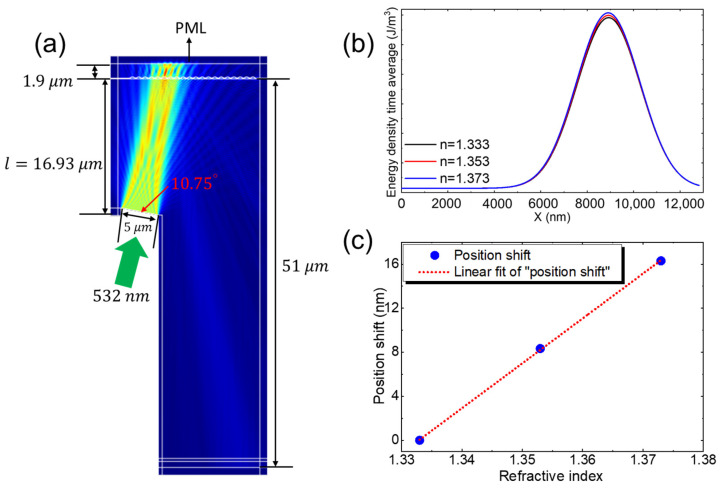
(**a**) Simulated field distribution with AuNPs placed above the glass substrate, with the grating positioned on the opposite side of the glass substrate. (**b**) Energy density time average with respect to x position at different refractive index and (**c**) Plot of the position shift versus the RI surrounding the AuNPs.

Numerical simulations were conducted to better understand the effect of local RI surrounding the AuNPs on the position shift of the diffracted spot in this PPR coupled diffraction grating waveguide sensing system. Finite element method (FEM) simulations were performed using COMSOL Multiphysics to evaluate the RIS. The RI of the solution sample varied from 1.333 to 1.373, while the grating’s RI was maintained at 1.53, the substrate at 1.73, and the AuNPs at 0.486 + 2.675i. The grating period and depth were set to 832 nm and 150 nm, respectively, with the AuNPs modeled at a diameter of 10 nm. The AuNPs were positioned above the glass substrate, with the grating on the opposite side, as illustrated in Figure 3a. A plane wave with a wavelength of 532 nm was incident on the grating at an angle of 19.96°, exciting the PPR modes within the system. Please note that, while performing the experiment, we adjusted the incident angle by rotating the sensor plate on a rotating stage rather than changing the incident angle of the light source to the sensor plate. Therefore, in the simulation model, we did not place the grating horizontally to ensure consistency with the experimental results. This approach allowed us to accurately replicate the conditions under which the particle plasmon modes were excited in the experiment. The glass slide facilitated the coupling of the plane wave at this incident angle, inducing a plasmonic grating resonant mode and dispersing the diffracted light. This method leverages the PPR without bulky optics. Due to the coupling of the PPR with diffraction, the angle of the diffracted beam becomes highly responsive to changes in the RI of the surrounding environment near the AuNP surface, as well as to biomolecular interactions occurring on the AuNP surface. Figure 3b shows the variation in time-averaged energy density with respect to the x-position at different RI varied from 1.333 to 1.373 in the simulated design, which mimics the actual fabricated structure of the sensor. The simulation results clearly demonstrate that as the RI increases, the energy density in the device also increases due to the PPR effect, which is sensitive to the local RI near the AuNP surface. This indicates that the position shift of the diffraction spot with AuNPs on the grating is sensitive to the RI around the AuNPs, making it useful for biomolecular detection.

To quantify the simulated RIS of the system, the positional shift of the diffracted beam was measured by exposing the bare AuNP surface to solutions with varying refractive indices. As the RI of the solution increased from 1.333 to 1.373, a linear shift in the diffracted beam position was noted, as depicted in Figure 3c. This demonstrates a simulated RIS of 0.72 mm/RIU, which reasonably aligns with the experimentally obtained RIS of 0.97 mm/RIU, validating the sensor’s efficacy and performance. The linear relationship shown in Figure 3c confirms the sensitivity of the system to changes in the RI around the AuNPs. The coupling between the PPR and diffraction significantly enhanced the sensitivity of the output field to RI variations near the AuNP surface. This sensitivity to environmental changes and biomolecular interactions on the AuNP surface highlights the potential of this approach for high-sensitivity biomolecular detection by leveraging the enhanced PPR effects of the diffraction-grating waveguide-coupled PPR system.

### 3.5. Biosensing Tests

**Figure 4:**
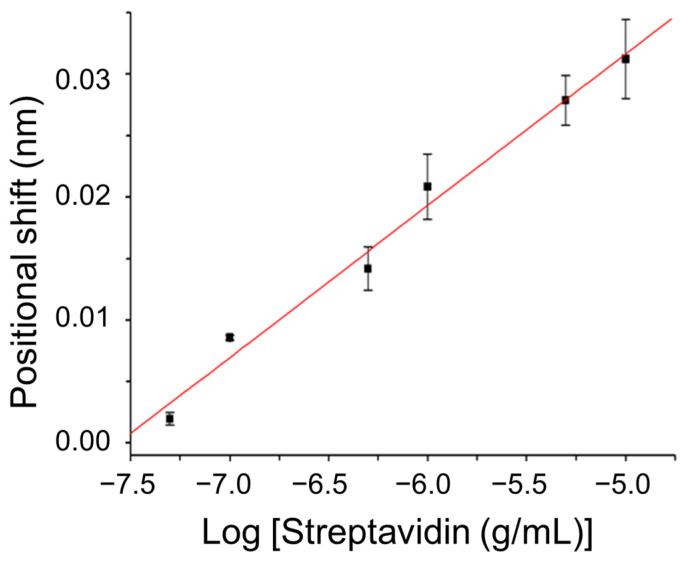
Position shift of diffracted beam at PSD versus logarithm of streptavidin concentration. Condition: thickness of glass slide = 1 mm.

To demonstrate the biosensing capability of the sensor, biomolecular interactions between the surface-immobilized biotin group and streptavidin were studied. According to a reported procedure, the biotin group was functionalized on the AuNP surface [6]. The progress of functionalization was monitored by analyzing spectral shifts in a self-assembled sub-monolayer of AuNPs on a glass slide, both before and after modification [36]. Streptavidin, which has a molecular mass of 60,000, binds to biotin with an apparent association constant of approximately 10^15^ M^−1^ [6]. We investigated the concentration-dependent responses of the biotin-functionalized sensor chip to assess its sensitivity for studying biotin–streptavidin binding. Figure 4 illustrates the calibration curve depicting the equilibrium sensor responses (position shifts) corresponding to streptavidin concentrations ranging from 5 × 10^−8^ to 1 × 10^−5^ g/mL. Across this concentration range, the plot illustrating the sensor response versus the logarithm of streptavidin concentration is linear (r = 0.993), as depicted in Figure 4. The limit of detection (LOD) is determined as the sensor response from a sample that yields a signal-to-noise ratio of 3, where the noise is calculated as the standard deviation of the diffracted beam position from three repeated measurements of a blank sample. The LOD of the sensor for streptavidin was 1.9 × 10^−8^ g/mL (3.2 × 10^−10^ M) using the 1 mm glass slide as the substrate. This LOD was significantly better than that of the counterpart biosensing approach with the PPR effect alone [3].

**Figure 5:**
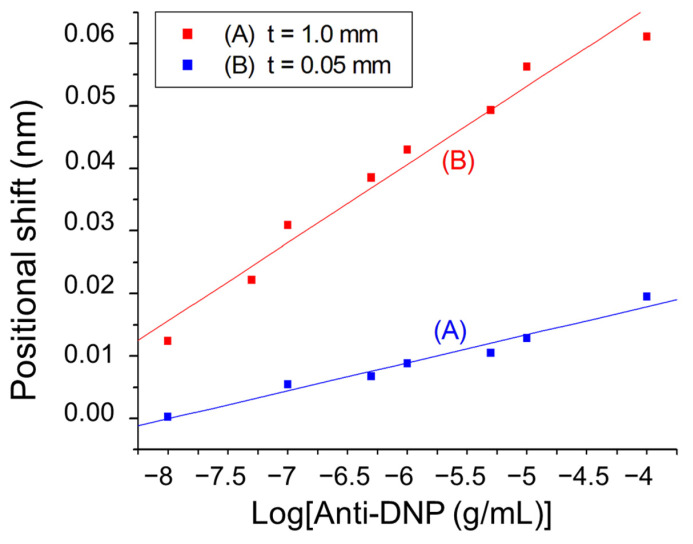
Plots of position shift of diffracted beam at PSD versus logarithm of anti-DNP concentration with (A) thickness of glass slide = 1 mm (*y* = 0.03588 + 0.00449 × log[*x*]), and (B) thickness of glass slide = 50 μm (*y* = 0.11568 + 0.01251 × log[*x*]), where *y* = position shift in mm and *x* = anti-DNP concentration in g/mL.

To further evaluate the effect of the glass substrate thickness on the biosensor sensitivity towards biomolecular detection, the biomolecular interactions between surface-immobilized dinitrophenyl (DNP) and anti-DNP antibodies was employed as a model. The DNP group was functionalized on the gold nanoparticle surface according to a reported procedure [37]. In this DNP–anti-DNP system, an anti-DNP antibody has a molecular mass of approximately 150,000 and binds a DNP antigen with an apparent association constant of approximately 1.8 × 10^6^ M^−1^ [38]. Over the anti-DNP concentration range from 1 × 10^−8^ to 1 × 10^−5^ g/mL, the plot of the sensor response using a 1 mm glass slide as the substrate versus log concentration of anti-DNP is linear (*r* = 0.9837), as shown in Plot A of Figure 5. From this plot, the LOD of the sensor for anti-DNP is determined to be 1.3 × 10^−8^ g/mL (5.8 × 10^−11^ M). A negative control was performed to test the specificity of the biosensor. As shown in Figure S11, the sensor responses towards anti-HSA over the same concentration range were negligibly small, indicating the excellent biological specificity of our biosensor. If a 50 μm glass slide was used as the substrate, the plot of the sensor response versus log concentration of anti-DNP is also linear (*r* = 0.9842), as shown in Plot B of Figure 5. Interestingly, the LOD was improved to 9.7 × 10^−10^ g/mL (4.4 × 10^−12^ M). The sensor sensitivity as defined by the slope of each calibration plot suggests that the sensor with the 1 mm glass slide substrate is more sensitive than that with the 50 μm glass slide substrate by 2.8 times, further confirming the advantage of using a thinner glass substrate in this sensor configuration. In comparison with the counterpart biosensing approach using a resonant grating waveguide alone, these LODs are significantly better [39]. Such LODs are also comparable or even better than the grating-based SPR sensors [22,40,41,42].

**Figure 6:**
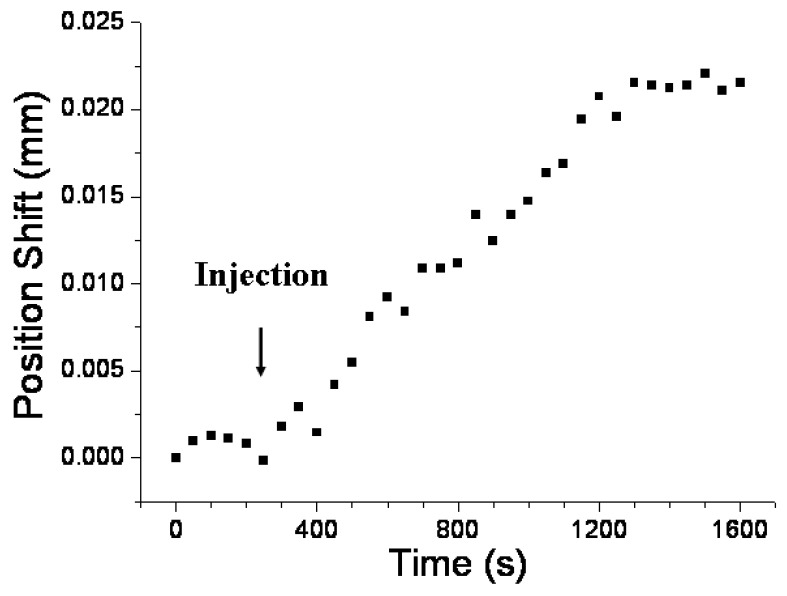
Temporal response of a DNP-functionalized sensor chip after injection of an anti-DNP solution (1 × 10^−4^ g/mL). Condition: Thickness of glass slide = 1 mm.

Real-time detection enables researchers to obtain immediate interactive information on a sample, such as association and dissociation kinetics [43,44]. The concentration-dependent responses of the sensor can be tracked in real time by monitoring the shift in position of the diffracted beam at the PSD. Figure 6 depicts the temporal sensor response of a DNP-functionalized sensor chip at an anti-DNP concentration of 1 × 10^−4^ g/mL. Upon injecting the anti-DNP solution, a notable change in sensor response occurred, stabilizing into a steady state after approximately 900 s. The time-dependent shift in the position of the diffracted beam at the PSD was attributed to the antigen–antibody interaction, causing a localized increase in the RI at the AuNP surface.

It should be noted that in this biosensor, the AuNPs are immobilized on a glass slide which is in contact with a sample, while the diffraction grating based on UV-assisted embossing is placed on the other side of the slide. Here, we name it as the opposite-side approach. Another possible arrangement is having the AuNPs and grating on the same side of a glass slide, i.e., with AuNP-modified grating in contact with a sample. Here, we name it as the same-side approach. Our results show that the same-side approach has similar or slightly better sensitivity as compared to that of the opposite-side approach. Performance comparison of both approaches is shown in Table 1. However, since the grating waveguide is in contact with a sample in the same-side approach, defects in the grating waveguide will affect the effective RI of the grating waveguide [45]. In particular, if a sample solution can infiltrate the defects, the effective RI of the grating waveguide will vary with the RI of the sample solution. This will cause a baseline shift of the sensor. Hence, the same-side approach requires better control in the grating fabrication processes to avoid defects for the sake of yielding more reproducible sensor plates. Furthermore, the same-side approach also needs functionalization of the grating surface for immobilization of AuNPs or recognition molecule to act as a sensor. This may be difficult for some grating materials. Therefore, the opposite-side approach provides a simple way to make a sensor.

## 4. Conclusions

In summary, the high sensitivity of this label-free biosensing platform presents a distinctive approach to analyzing biointeractions. It utilizes a straightforward and cost-effective optical setup with disposable chips and requires only a small sample volume. The grating-coupled PPR sensor chip shows promise in scalability to a laser spot without sacrificing sensitivity and can be integrated with microfluidics for high-throughput analysis. Ongoing efforts in our laboratory are focused on integrating multiple gratings into a microarray format for multiplexed biosensing applications.

## Figures and Tables

**Table 1:** Performance comparison between the opposite-side and same-side approaches.

Figure-of-Merit	Opposite-Side	Same-Side
SR (*t =* 1.0 mm)	7.8 × 10^−4^ RIU	8.3 × 10^−4^ RIU
LOD for ant-DNP (*t* = 1.0 mm)	5.8 × 10^−11^ M	3.2 × 10^−11^ M
LOD for ant-DNP (*t* = 0.05 mm)	4.4 × 10^−12^ M	1.4 × 10^−12^ M

## Data Availability

The data supporting this article are included in Supplementary Information. The raw data supporting the findings of this study are available from the corresponding author upon reasonable request.

## Supplementary Materials

The following supporting information can be downloaded at: https://www.mdpi.com/article/10.3390/s24175536/s1, Figure S1: Photograph of microfluidic sensor chip; Figure S2: UV–vis absorption spectrum of AuNP solution; Figure S3: TEM image and size distribution of AuNPs. Figure S4. Photograph of PDMS stamp; Figure S5: AFM image and image analysis of PDMS stamp; Figure S6: AFM image and image analysis of the sol–gel grating; Figure S7. FE-SEM image of sol–gel grating; Figure S8: IR spectra of sol–gel film; Figure S9: Cross-sectional FE-SEM micrograph of sol–gel film; Figure S10: Plots of intensity decay versus propagation distance through sol–gel films; Figure S11. Specificity tests.
