# Supplementary material for: Label-Free Biosensor Based on Particle Plasmon Resonance Coupled with Diffraction Grating Waveguide
Source: Sensors (Basel). 2024 Aug 27;24(17):5536. doi: 10.3390/s24175536 (PMC11397741; doi:10.3390/s24175536)
Supplement: Supplementary file 1 [file sensors-24-05536-s001.zip › sensors-3135404-supplementary.pdf]

SUPPLEMENTARY INFORMATION

Label-Free Biosensor Based on Particle Plasmon Resonance Coupled with Diffraction Grating Waveguide

Wei-Ting Hsu [1], Yu-Cheng Lin [2], Huang-Chin Yang [1], Devesh Barshilia [2], Po-Liang Chen [2], Fu-Chun Huang [2], Lai-Kwan Chau [1,*], Wen-Hsin Hsieh [2,*] and Guo-En Chang [2,*]

[1]Department of Chemistry and Biochemistry and Center for Nano Bio-Detection, National Chung Cheng University, Chiayi 621301, Taiwan
[2]Department of Mechanical Engineering and Advanced Institute of Manufacturing with High-tech Innovations, National Chung Cheng University, Chiayi 621301, Taiwan
*E-mails: chelkc@ccu.edu.tw (L.-K. Chau), imewhh@ccu.edu.tw (W.-H. Hsieh), imegec@ccu.edu.tw (G.-E. Chang)

TABLE OF CONTENTS

Figure S1. Photograph of microfluidic sensor chip.
Figure S2. UV–Vis absorption spectrum of AuNP solution.
Figure S3. TEM image and size distribution of AuNPs.
Figure S4. Photograph of PDMS stamp.
Figure S5. AFM image and image analysis of PDMS stamp.
Figure S6. AFM image and image analysis of sol-gel grating.
Figure S7. FE-SEM image of sol-gel grating.
Figure S8. IR spectra of sol-gel film.
Figure S9. Cross-sectional view of FE-SEM micrograph of sol-gel film.
Figure S10. Plots of intensity decay versus propagation distance through sol-gel films.
Figure S11. Specificity tests.

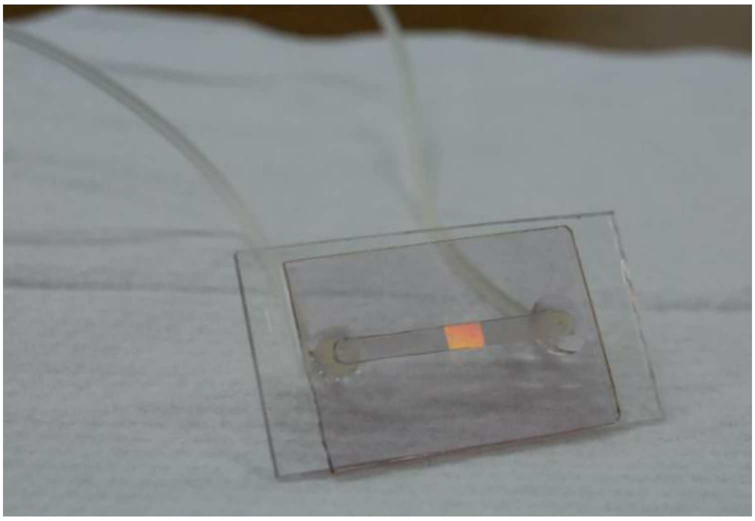

Figure S1. A photograph of the microfluidic sensor chip.

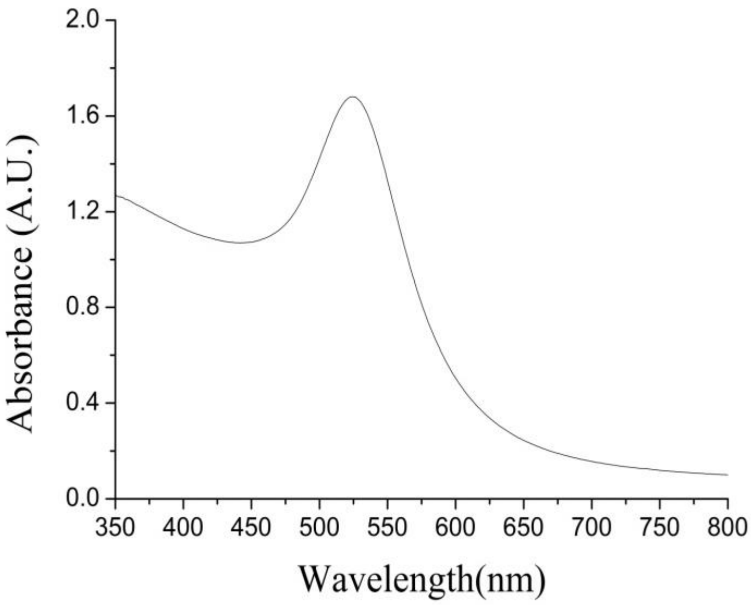

Figure S2. UV–Vis absorption spectrum of a solution of AuNPs in aqueous medium.

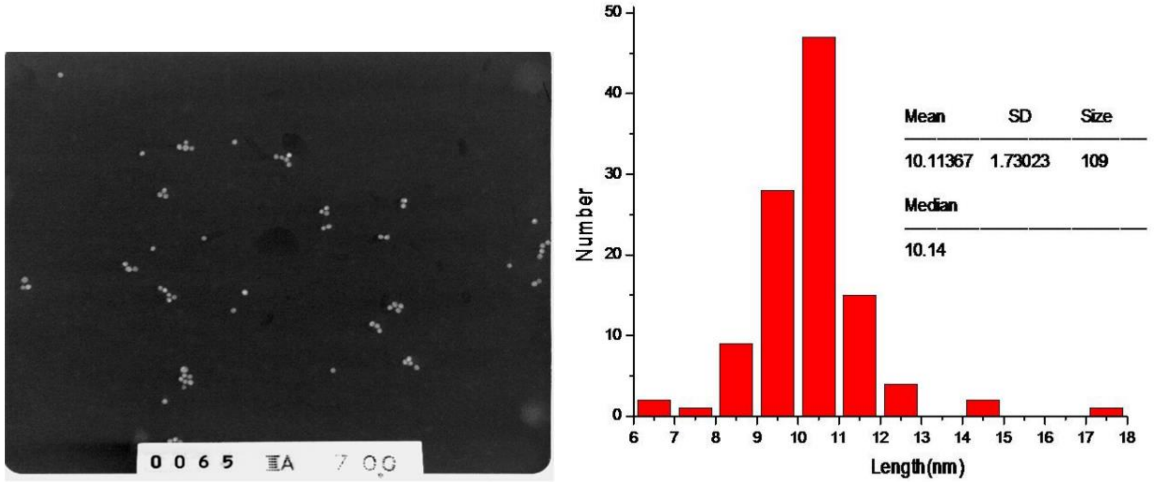

(a) (b)

Figure S3. (a) TEM image of AuNPs. (b) Size distribution of AuNPs by TEM image analysis. Average diameter = 10.1 ± 1.7 nm (n = 100).

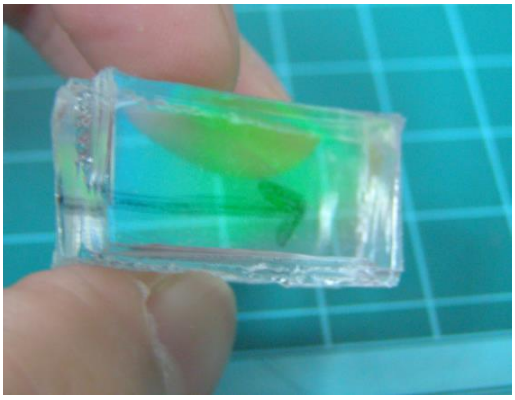

Figure S4. A photograph the PDMS stamp after detaching from the holographic glass grating (master grating).

(a)

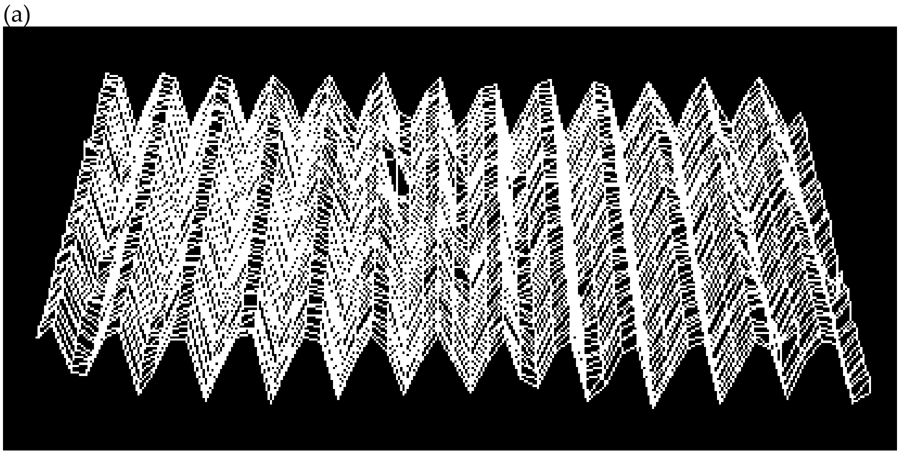

(b)

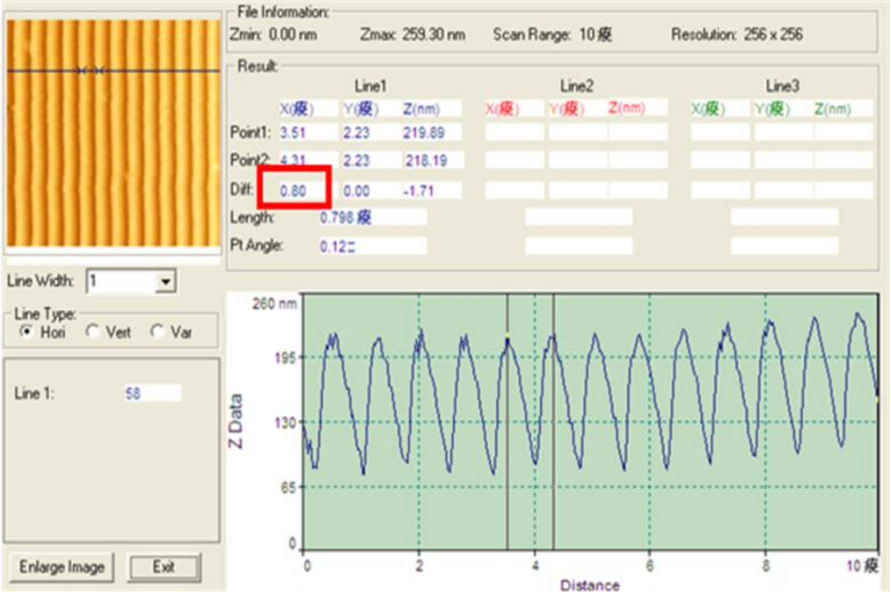

(c)

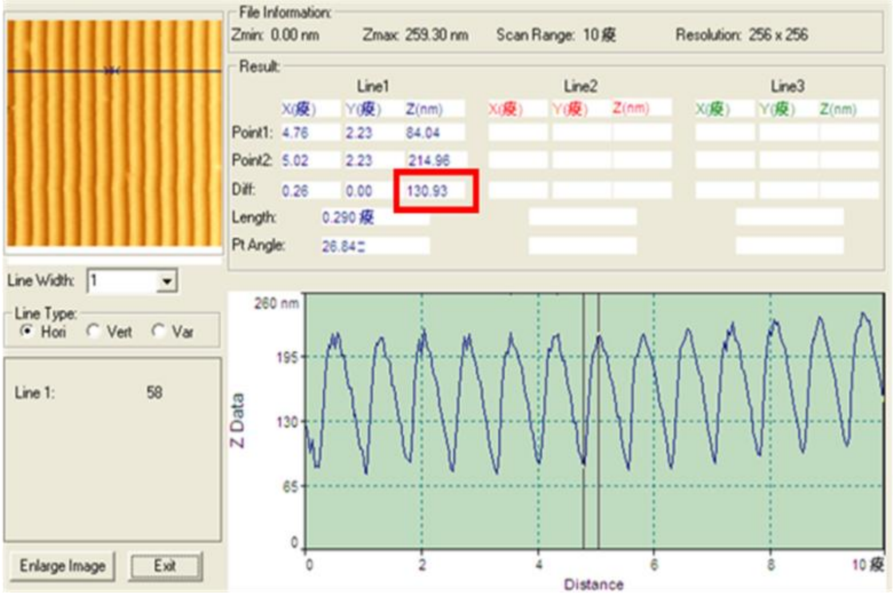

Figure S5. (a) AFM image of a PDMS stamp. (b) AFM image analysis of the period of the grating structure, and (c) AFM image analysis of the depth of the grating structure.

(a)

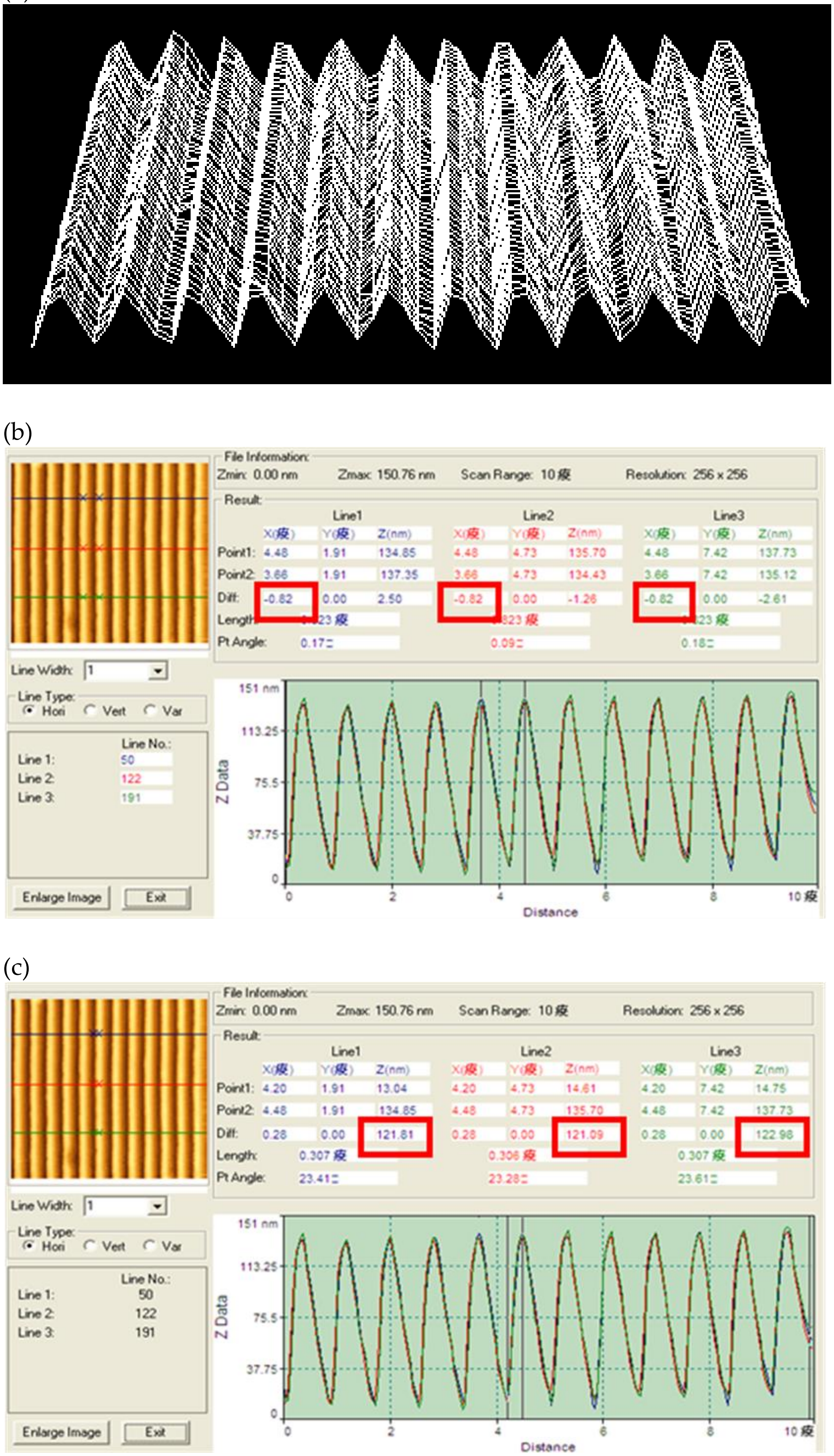

Figure S6. (a) AFM image of a sol-gel grating. (b) AFM image analysis of the period of the grating structure, and (c) AFM image analysis of the depth of the grating structure.

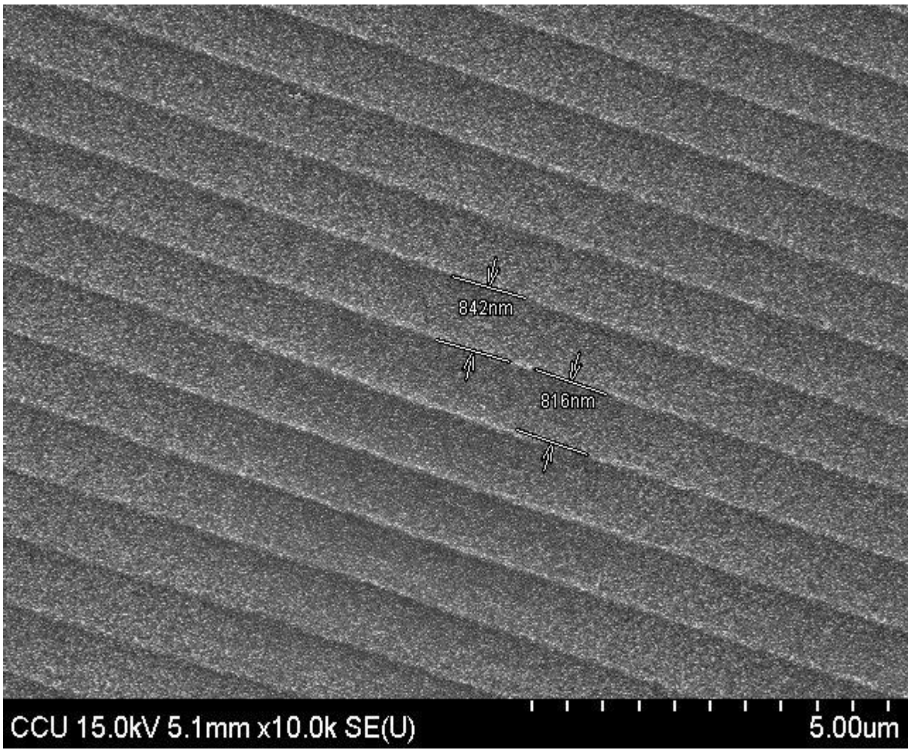

Figure S7. FE-SEM image of a sol-gel grating.

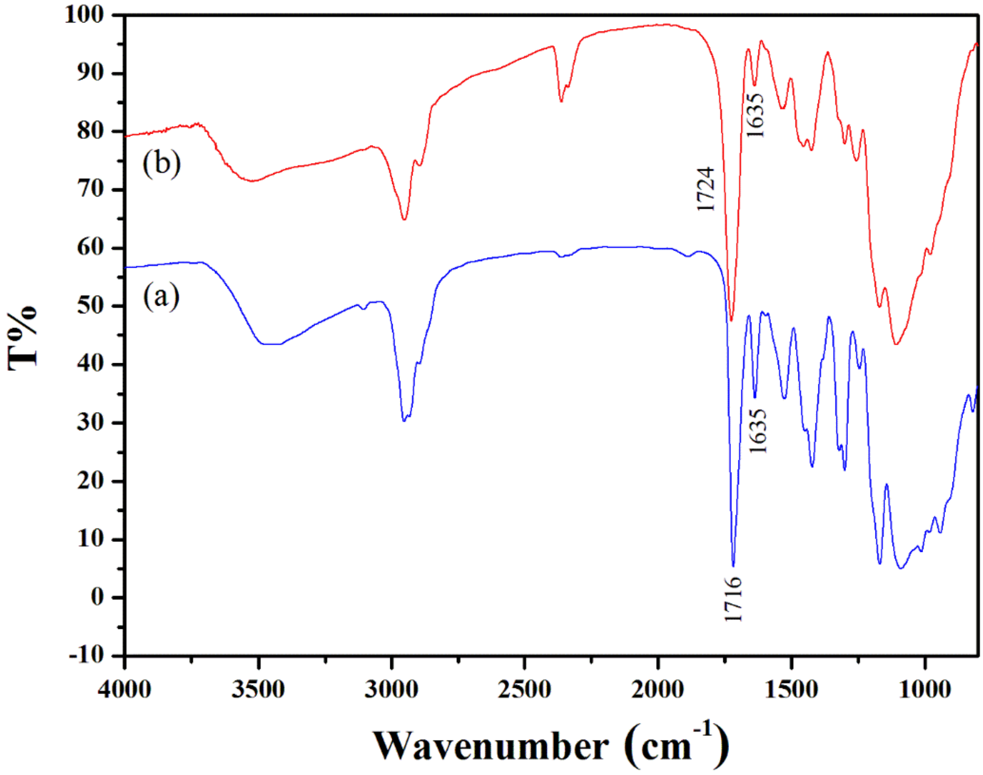

Figure S8. IR spectra of a sol-gel film (a) before and (b) after exposure to UV light. Note: Figure (a) is offset in y-axis to aid visualization.

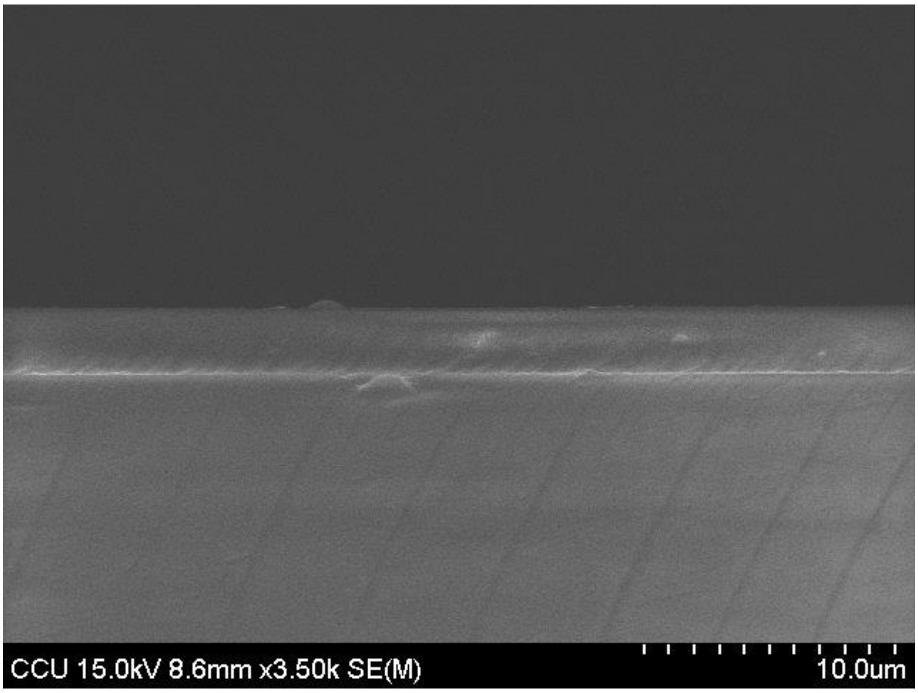

Figure S9. Cross-sectional view of the FE-SEM micrograph of a sol-gel film.

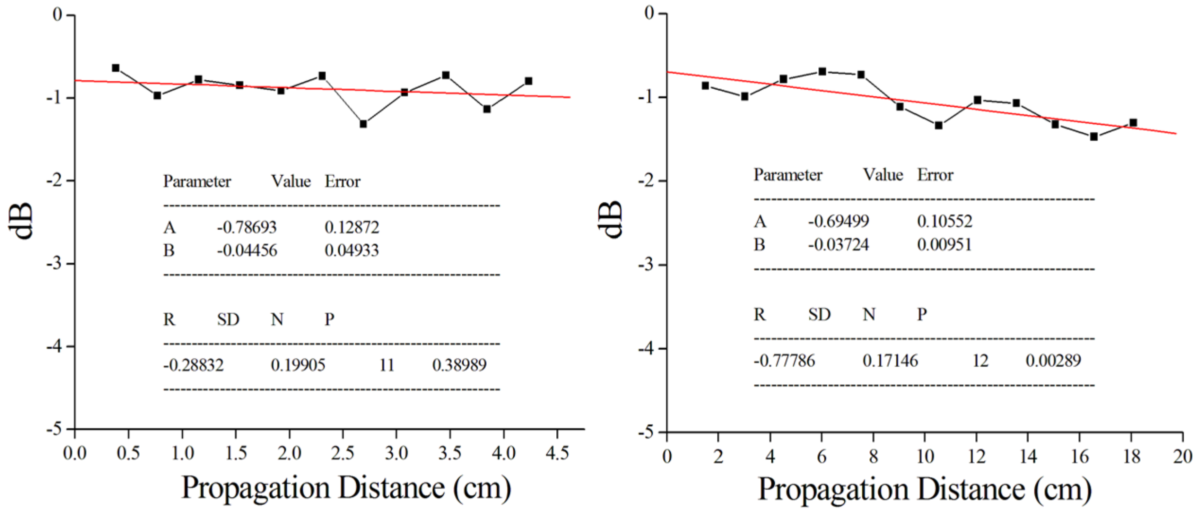

Figure S10. Plots of intensity decay versus propagation distance through two sol-gel films.

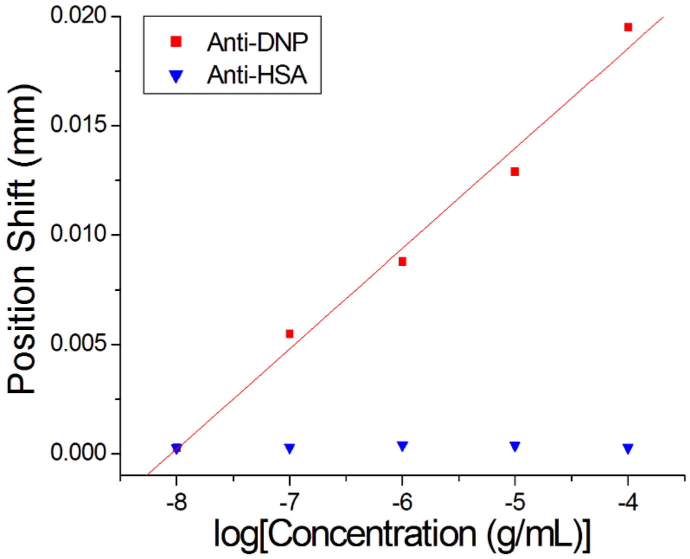

Figure S11. Specificity tests: Plots of position shift of diffracted beam at PSD versus (a) logarithm of anti-DNP concentration (red square) and (b) logarithm of anti-HSA concentration (blue triangle).
